# The Endoscopic Removal of Eroded Foreign Bodies in the Ureter

**DOI:** 10.1089/cren.2016.0009

**Published:** 2016-02-01

**Authors:** David Anthony Kurz, Phillip Mucksavage

**Affiliations:** Division of Urology, Department of Surgery, University of Pennsylvania, Philadelphia, Pennsylvania.

## Abstract

A complication of using foreign materials in surgery is potential erosion into nearby tissues. The endoscopic removal of foreign bodies that have eroded into the urinary tract is a safe and minimally invasive option that has previously been described, most commonly in the bladder and urethra. We present the case of a patient who had a remote history of a pyeloplasty and was found to have different foreign bodies eroding into the ureter causing symptoms. To our knowledge, this is the first case where a patient presented with two different types of ureteral foreign body erosions that were each effectively treated endoscopically.

## Introduction

Iatrogenic foreign bodies in the urinary tract are a well-described phenomenon. Materials such as mesh, suture, Hem-o-Lok^®^ (Teleflex Medical, Research Triangle Park, NC) clips, and staples have the potential to erode into the surrounding tissue and have been previously reported in the literature.^1–4^ In the proper setting, symptoms such as pain, urinary tract infections, and lower urinary tract symptoms (i.e., frequency, hematuria, and dysuria) may be a harbinger for foreign body erosion into the urinary tract. This is often a challenging dilemma for clinicians and can ultimately necessitate invasive treatment depending on both the location and degree of erosion, along with patient-related factors. Another option for removing these foreign bodies includes using a minimally invasive endoscopic approach. We present the case of a patient with a remote history of a pyeloplasty followed by two separate instances of foreign body erosion into the ureter at the ureteropelvic junction (UPJ) that were removed endoscopically.

## Case Presentation

**FIG. 1.**
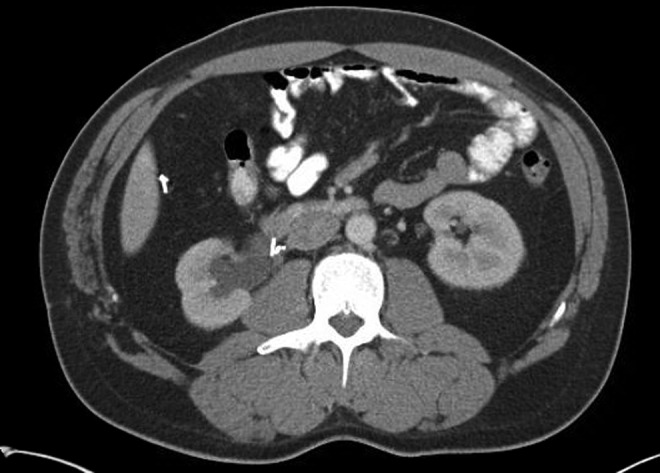
CT of the abdomen without contrast showing surgical clips near the ureteropelvic junction (UPJ). From this image, it is difficult to tell whether the clips are eroding into the ureter.

A 38-year-old male with a history of an open right pyeloplasty in 1997 for a right UPJ obstruction presented 18 years later with intermittent right flank pain that he had been having since the initial operation as well as hematuria. His urine culture was positive for *Klebsiella pneumonia* and he was treated for pyelonephritis. A CT of the abdomen without contrast at that time showed moderate right intrarenal caliectasis with a dilated renal pelvis. Multiple surgical clips were seen at the right UPJ. There were no calculi seen (Fig. 1). A renal scan showed 50% function from both the right and left kidneys. The right kidney had both normal blood flow and cortical uptake with an adequate excretion. There was significant tracer retention on the right side that responded appropriately to Lasix administration.

**FIG. 2.**
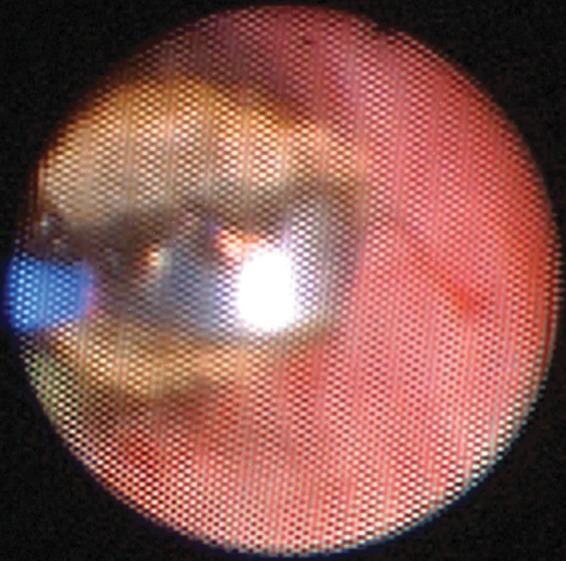
Calcified metal clip showing lasering of the encrustation.

He elected to undergo a cystoscopy and right ureteroscopy to evaluate a new or persistent UPJ obstruction. The flexible ureteroscope was able to be advanced into the dilated right renal pelvis. The ureteroscope was withdrawn and an encrusted metal surgical clip was seen at the UPJ that was embedded in the wall of the ureter. The encrustation was lasered with a holmium laser fiber and the stone fragments and clip were retrieved with a basket (Fig. 2). No other foreign bodies were found either in the kidney or in the ureter. A ureteral stent was placed and removed uneventfully several days later.

**FIG. 3.**
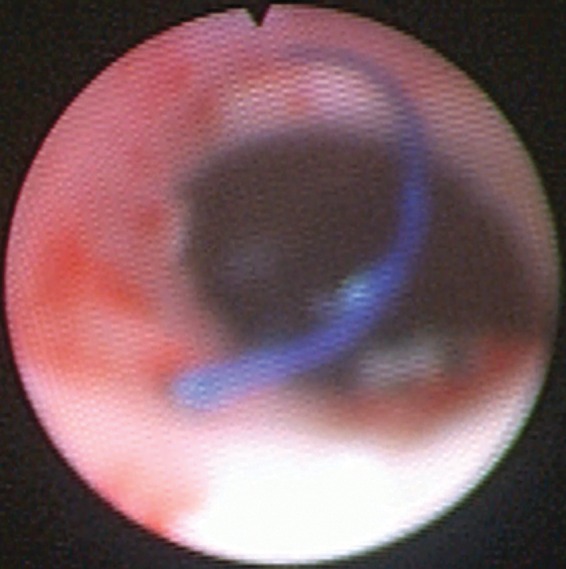
Suture material seen eroding into the UPJ and proximal ureter.

His symptoms completely resolved for 5 months after the procedure, until he had acute onset hematuria, dysuria, and right flank pain—which were the same symptoms he had before the clip removal. A repeat ureteroscopy was recommended to evaluate for additional eroded foreign bodies. Ureteroscopy revealed suture material eroding through the lumen of the ureter just distal to the UPJ. A basket was used to pull as much of the suture as possible into the lumen. Next, a 272 μm holmium laser fiber was used at 0.2 J and 10 Hz to sever the intraluminal suture. This fragment was then grasped and removed with a basket (Fig. 3). There was no exposed suture remaining. Gross inspection of the specimen revealed a 1 cm piece of suture material. He remains asymptomatic at follow-up at 6 months.

## Discussion and Review of the Literature

The erosion of foreign bodies into the upper urinary tract has been shown to occur with partial nephrectomy and pyeloplasty.^5–10^ Previous reports describe the presentation of symptoms between 6 weeks and 2 years after the initial procedure.^5–6^ In this case, the patient reported symptoms shortly after surgery, but he did not undergo an endoscopic examination until many years later when his pain worsened and he developed new symptoms (hematuria and urinary tract infections). Other commonly reported symptoms are flank/abdominal pain, fever, nausea, vomiting, and lower urinary tract symptoms.

It is unclear what causes the migration into the collecting system, but it has been postulated to be related to surgery involving entry into the urinary tract (either deliberate or inadvertent) or placing foreign bodies in proximity to the collecting system.^7^ Finley and colleagues describe the case of a laparoscopic pyeloplasty complicated by anastomotic extravasation, bacteriuria, and funguria with a large pseudodiverticulum containing numerous Lapra-Ty suture clips (Ethicon Endosurgery, Cincinnati, OH).^8^ This suggests that erosion may be more likely with certain postoperative conditions such as poor wound healing, infection, hematomas, and urinomas.

Foreign bodies themselves can resemble a stone on imaging, or they may in fact be encased in calcifications.^9,10^ As a result, the diagnosis is difficult to ascertain on the basis of imaging alone. Most often, they can be diagnosed and treated endoscopically with either ureteroscopy or percutaneous nephroscopy. This allows for stone or suture lasering if present as well as a minimally invasive means of removing small foreign bodies.

Our patient presented nearly two decades after his initial procedure and was found to have a completely eroded metal clip into the ureter. This acted as a nidus for stone formation, and when removed, his symptoms resolved. Several months later, newly eroded suture material (likely Prolene) in the same area of the ureter was found to be producing the identical symptoms as seen previously. Both of these foreign bodies were removed endoscopically. To our knowledge, this is the first reported case of different eroded foreign surgical materials removed endoscopically from the ureter on two separate occasions nearly two decades after open pyeloplasty.

## Conclusion

Collecting system erosion of surgical material in some situations can be difficult to diagnose; however, one should consider the possibility in patients who have had previous surgery involving or near the urinary tract. When feasible, an endoscopic approach to their removal can be safe and effective.

## Abbreviations Used

CT: computed tomography
UPJ: ureteropelvic junction
